# The effect of task load, information reliability and interdependency on anticipation performance

**DOI:** 10.1186/s41235-024-00548-8

**Published:** 2024-04-14

**Authors:** Colm P. Murphy, Oliver R. Runswick, N. Viktor Gredin, David P. Broadbent

**Affiliations:** 1 Cardiff School of Sport and Health Sciences, Cardiff Metropolitan University, Cardiff, CF23 6XD UK; 2 Faculty of Sport, Technology and Health Sciences, St. Mary’s University, Twickenham, UK; 3 Department of Psychology, Institute of Psychiatry, Psychology & Neuroscience, King’s College London, London, UK; 4 School of Health and Welfare, Halmstad University, Halmstad, Sweden; 5 Centre for Cognitive Neuroscience, College of Health, Medicine and Life Sciences, Brunel University London, London, UK; 6 Centre for Sport Research, Institute for Physical Activity and Nutrition, Deakin University, Burwood, VIC Australia

**Keywords:** Cognitive load, Contextual information, Kinematic information, Perceptual-cognitive expertise, Sport

## Abstract

In sport, coaches often explicitly provide athletes with stable contextual information related to opponent action preferences to enhance anticipation performance. This information can be *dependent* on, or *independent* of, dynamic contextual information that only emerges during the sequence of play (e.g. opponent positioning). The interdependency between contextual information sources, and the associated cognitive demands of integrating information sources during anticipation, has not yet been systematically examined. We used a temporal occlusion paradigm to alter the reliability of contextual and kinematic information during the *early, mid-* and *final* phases of a two-versus-two soccer anticipation task. A dual-task paradigm was incorporated to investigate the impact of task load on skilled soccer players’ ability to integrate information and update their judgements in each phase. Across conditions, participants received no contextual information (*control*) or stable contextual information (opponent preferences) that was *dependent* on, or *independent* of, dynamic contextual information (opponent positioning). As predicted, participants used reliable contextual and kinematic information to enhance anticipation. Further exploratory analysis suggested that increased task load detrimentally affected anticipation accuracy but only when both reliable contextual and kinematic information were available for integration in the final phase. This effect was observed irrespective of whether the stable contextual information was dependent on, or independent of, dynamic contextual information. Findings suggest that updating anticipatory judgements in the final phase of a sequence of play based on the integration of reliable contextual and kinematic information requires cognitive resources.

**Supplementary Information:**

The online version contains supplementary material available at 10.1186/s41235-024-00548-8.

## Significance statement

Technological advancements in sports performance analysis have resulted in coaches having access to a range of information about the action preferences of upcoming opponents; for example, the tendency of opposition players to pass to teammates or attempt to dribble past defenders. Invariably, coaches explicitly provide this information to their athletes prior to competition, aiming to promote performance gains through enhanced anticipation. However, using this contextual information effectively in competition requires cognitive resources, particularly when required to update the information with dynamically evolving environmental information (e.g. opponent/teammate positioning) and integrate it with kinematic cues from the oncoming opponent. Given the cognitive demands placed on athletes in competition, and the limited working memory resources of humans, it is necessary to investigate how effectively skilled athletes use this information under increased task demands. In this study, a dual-task paradigm was used to explore the proposal that utilising different types of contextual information regarding opponent action preferences can be more or less cognitively demanding based on its dependence on dynamically evolving information related to teammate positioning and the phase of an action. Dual-task conditions reduced anticipatory performance at phases of the action which required integration of contextual and kinematic information. Coaches should therefore consider the potential cognitive load experienced by athletes in a given sport or situation, when deciding what type of information to provide them to enhance their anticipation of future opponents.

## Introduction

The ability to use contextual and kinematic information to facilitate predictive judgements is a significant marker of expertise in sport (Loffing & Cañal-Bruland, 2017; Müller & Abernethy, 2012; Williams & Jackson, 2019). Kinematic information emanates from biological motion of the opponent and becomes more reliable in the final phases of an unfolding action (e.g. Farrow et al., 2005; Jones & Miles, 1978). Contextual information broadly refers to any non-kinematic information that is relevant to a specific situation or domain (Runswick et al., 2020). Contextual information can be viewed as stable or dynamic and is used to inform the athlete’s early anticipatory judgements until integrated and updated with reliable kinematic information (Gredin et al., 2020a). Stable contextual sources of information are established before an event commences (e.g. a priori information about an opponent’s action preferences), with the reliability of this information being contingent on its interdependency with dynamic contextual information that emerges as the event unfolds (e.g. the evolving positioning of an opponent). It has been suggested that the use of *dependent* and *independent* stable contextual information evokes different levels of cognitive load (e.g. Gredin et al., 2020b; Runswick et al., 2018). However, this proposition, and the subsequent effect of various levels of task load on anticipation performance, is yet to be examined in a systematic manner (Gredin et al., 2020a). The aim of the current study was to systematically examine the impact of increased task load on the use of stable contextual information (i.e. opponent action preferences), that is *dependent* on, or *independent* of, dynamic contextual information (i.e. opponent positioning), as well as the integration of this information with kinematic cues, during an evolving two-versus-two video-based soccer anticipation task.

There is a growing body of empirical evidence for the use of Bayesian integration theory as a framework for anticipation in sport (e.g. Gredin et al., 2018, 2020c; Harris et al., 2022; Helm et al., 2020; Loffing & Hagemann, 2014). Bayesian theory suggests that individuals make predictive judgements based on causal probabilistic relationships (i.e. probabilistic if–then relationships) between known information variables and unknown to-be-anticipated variables (Körding, 2007). If one information variable is associated with greater reliability (i.e. lower uncertainty) than another, then the individual’s predictive judgement should be biased towards the more reliable information (Knill & Pouget, 2004). In other words, the individual’s reliance on various sources of information is contingent upon the comparative reliability of the information, with greater weight assigned to information of higher reliability (Vilares & Körding, 2011).

In sport, advances in technology have enabled sophisticated performance analyses of opponents, and athletes are often explicitly primed with information about upcoming opponents to enhance in-match anticipation and decision making (Memmert et al., 2017). However, assuming athletes use domain-specific knowledge to make predictive judgements based on conditional inference and application of probabilistic if–then relationships, it could be argued that increasing the number of probabilistic rules through the provision of explicit contextual information may lead to increases in cognitive load (Waldmann & Hagmayer, 2001). Furthermore, reliance on such explicit a priori contextual information induces a top-down, context-driven selection of unfolding environmental information (Gredin et al., 2018), which is associated with greater processing demands than bottom-up, or stimulus-driven, attentional processes (Kaplan & Berman, 2010).

Since working memory capacity is limited (Paas et al., 2003), it is important to investigate the cognitive load associated with the process of updating and integrating contextual and kinematic information during anticipation. However, the literature reveals contrasting findings as to whether the processes involved in anticipation draw on the performer’s limited working memory resources. Runswick et al. (2018) examined the impact of dual-task conditions (i.e. a backward-counting secondary task) on the use of stable contextual information in a cricket batting anticipation task. The addition of stable contextual information related to the game state and field setting did not alter perceived cognitive load of batters and anticipation performance was actually better in the dual-task condition. The authors suggested that integrating contextual and kinematic information may be governed by automatic processes and the secondary task supressed conscious control, thus enhancing these processes (Runswick et al., 2018). In contrast, Gredin et al. (2020b) observed that the explicit provision of stable contextual information related to the opponent action preferences increased players’ cognitive load during a soccer anticipation task (see also, Simonet et al., 2019). Moreover, the implementation of a secondary *n* back task, diminished the performance enhancing effects of stable contextual priors observed under single-task conditions. The authors speculated that the contradictory findings compared to the study by Runswick et al. (2018) could be due to the difference in the *interdependency* of the sources of contextual information examined in the two studies.

Much of the previous research has provided participants with stable contextual information that is *independent* of dynamic contextual information and can be used reliably from the outset of the action (e.g. Broadbent et al., 2018; Murta et al., 2021; Navia et al., 2013; Runswick et al., 2018). In contrast, stable contextual information that is *dependent* on dynamic contextual information can only be reliably used once it is updated with dynamic contextual information that emerges as the event unfolds (Gredin et al., 2020a). Gredin et al. (2020b) utilised a two-versus-two video-based soccer anticipation task, developed by Gredin et al. (2018), that provides a unique insight into the interdependency between *stable* and *dynamic* contextual information. In the task, participants take the role of the defender and are required to predict the ball direction (left or right) following the final action from an opponent. Stable contextual information regarding the tendencies of the opponent can be explicitly provided to the participants, but importantly, this information can either be related to the opponents’ *action type* preference (i.e. % pass/dribble) or *directional* preference (i.e. % left/right), which changes the interdependency between stable and dynamic sources of contextual information. To reliably use information related to the opponents’ action type preference, the participant must know the direction of the potential pass, and so would need to process dynamic contextual information related to the positioning of the opposition teammate when it emerges during the action sequence (e.g. Gredin et al., 2018, 2020b). In contrast, directional preferences can be reliably used from the start of an action regardless of other information sources during the event (e.g. Broadbent et al., 2018). Thus, information about the attacker’s *directional* preferences is considered *independent* of any dynamic contextual information, whereas information about the attacker’s *action type* preferences is *dependent* on dynamic contextual information. To effectively utilise interdependent sources of stable and dynamic contextual information, the additional monitoring and updating processes may require increased cognitive resources, resulting in a decrement in performance under dual-task conditions, but a more systematic examination of these processes is required (Gredin et al., 2020a, 2023).

**Fig. 1:**
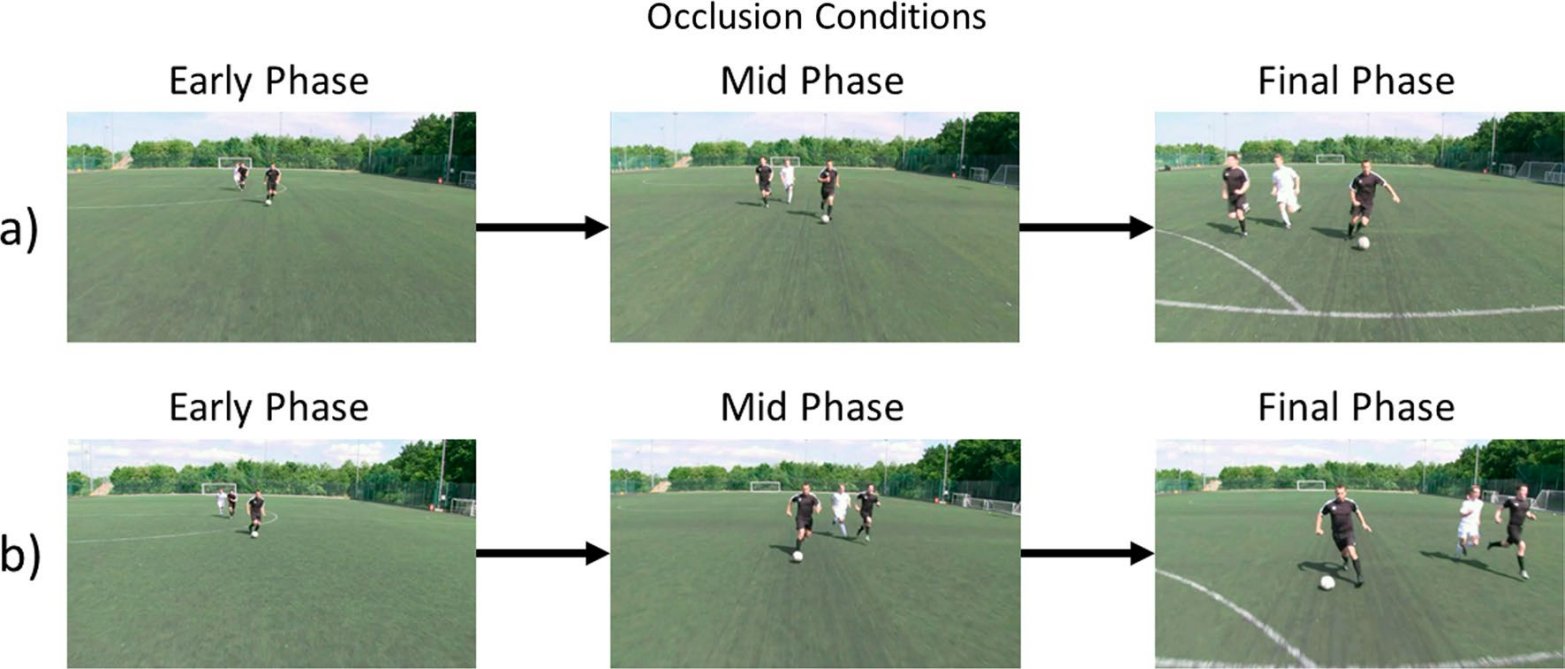
A schematic of the final frame of each occlusion condition (i.e. early phase [1 s], mid-phase [3 s] and final phase [5 s]). Rows a) and b) highlight the two actions the opponent off-the-ball could perform during a trial; **a** sequences in which the opponent off-the-ball stays on the same side they began their run on and **b** sequences in which the opponent off-the-ball crosses over to finish on the other side of the opponent in possession. Note: the outcome in both examples below was a dribble by the opponent in possession

The aim of the current study is to investigate how changes in information reliability and task load affect the interplay between stable contextual information, dynamic contextual information and kinematic information during anticipation in soccer. The same anticipation task developed by Gredin and colleagues (Gredin et al., 2018, 2020b, 2020c) was adopted in the current study, in which skilled soccer players were required to anticipate the direction (left or right) of the opponent’s final action in a defensive two-versus-two scenario. Trials were occluded in three phases (early phase, mid-phase and final phase; Fig. 1, Table 1) and had three context conditions related to the explicit stable contextual information provided to the participants: *Control* (i.e. no stable contextual information provided), *Dependent* (i.e. opponent action type preferences [% Dribble vs Pass] provided) and *Independent* (i.e. opponent directional preferences [% Left vs Right] provided). In each combined occlusion and context condition, participants performed the primary anticipation task under single- and dual-task conditions. A dual-task paradigm (letter recall) was implemented to examine the impact of task load on the updating and integration processes.

**Table 1:** A description of the nine experimental conditions based on the manipulations of the three independent variables (i.e. Occlusion condition, Context condition, Task Load condition)

Occlusion condition	Context condition	Task load condition	Description	Reliable information
Early phase	Independent	Single-Task (no secondary task)	Stable contextual information pertaining to the directional preferences of the opponent (Right = 67%, Left = 33%) was explicitly provided. This information was not contingent on other sources of contextual information. Footage occluded at 1 s, before the player off-the-ball initiated a movement to either side of the opponent in possession	• Stable Contextual Information (directional preferences)
		Dual-Task (letter recall task)		
	Dependent	Single-Task (no secondary task)	Stable contextual information pertaining to the action type preferences of the opponent (Dribble = 67%, Pass = 33%) was explicitly provided, which required the pickup of dynamic contextual information (i.e. position of the opponent off-the-ball) to interpret it. Footage occluded at 1 s, before the opponent off-the-ball initiated a movement to either side of the opponent in possession	
		Dual-Task (letter recall task)		
	Control	Single-Task (no secondary task)	No stable contextual information was explicitly provided in this condition. Footage occluded at 1 s, before the opponent off-the-ball initiated a movement to either side of the opponent in possession	
		Dual-Task (letter recall task)		
	Independent	Single-Task (no secondary task)	Stable contextual information pertaining to the directional preferences of the opponent (Right = 67%, Left = 33%) was explicitly provided. This information was not contingent on other sources of contextual information. Footage occluded at 3 s, after the opponent off-the-ball performed a movement to either side of the opponent in possession	• Stable Contextual Information (directional preferences)
		Dual-Task (letter recall task)		
Mid-phase	Dependent	Single-Task (no secondary task)	Stable contextual information pertaining to the action type preferences of the opponent (Dribble = 67%, Pass = 33%) was explicitly provided, which required the pickup of dynamic contextual information (i.e. position of the opponent off-the-ball) to interpret it. Footage occluded at 3 s, after the opponent off-the-ball performed a movement to either side of the opponent in possession	• Stable Contextual Information (action type preferences) • Dynamic Contextual Information (opponent positioning)
		Dual-Task (letter recall task)		
	Control	Single-Task (no secondary task)	No stable contextual information was explicitly provided in this condition. Footage occluded at 3 s, after the opponent off-the-ball performed a movement to either side of the opponent in possession	
		Dual-Task (letter recall task)		
Final phase	Independent	Single-Task (no secondary task)	Stable contextual information pertaining to the directional preferences of the opponent (Right = 67%, Left = 33%) was explicitly provided. This information was not contingent on other sources of contextual information. Footage occluded at 5 s, after the opponent off-the-ball performed a movement to either side of the opponent in possession and just before the opponent in possession carried out the final action (i.e. kinematic information was available)	• Stable Contextual Information (directional preferences) • Kinematic Information
		Dual-Task (letter recall task)		
	Dependent	Single-Task (no secondary task)	Stable contextual information pertaining to the action type preferences of the opponent (Dribble = 67%, Pass = 33%) was explicitly provided, which required the pickup of dynamic contextual information (i.e. position of the opponent off-the-ball) to interpret it. Footage occluded at 5 s, after the opponent off-the-ball performed a movement to either side of the opponent in possession and just before the opponent in possession carried out the final action (i.e. kinematic information was available)	• Stable Contextual Information (action type preferences) • Dynamic Contextual Information (opponent positioning) • Kinematic Information
		Dual-Task (letter recall task)		
	Control	Single-Task (no secondary task)	No stable contextual information was explicitly provided in this condition. Footage occluded at 5 s, after the opponent off-the-ball performed a movement to either side of the opponent in possession and just before the opponent in possession carried out the final action (i.e. kinematic information was available)	• Kinematic Information
		Dual-Task (letter recall task)		

The reliable sources of information available in each of the combinations of occlusion and context conditions are highlighted in the final column. Note: a blank cell in the Reliable Information column means there was no reliable information available that could be used by the participant to anticipate the final action

We hypothesised that when viewing the early phase of the action sequence, anticipation accuracy would only be enhanced in the *Independent* condition as the stable contextual information related to the opponent’s directional preference was the only source of information that was reliable at this stage of the trial. In the mid-phase, the dynamic contextual information related to the positioning of the opponent off-the-ball became reliable, and so, the stable contextual information related to the opponent’s action type preference in the *Dependent* condition could be utilised, meaning that, compared to the *Control* condition, performance would be enhanced in both the *Independent* and *Dependent* condition. In the final phase, kinematic information became reliable and could be used across all conditions, but we hypothesised that performance would be at its highest in the two stable contextual information conditions, as participants could integrate and update the contextual information with reliable kinematic information (Gredin et al., 2018, 2020c; Müller & Abernethy, 2012). We also hypothesised performance to be impacted differently by the dual-task depending on the combined occlusion and context condition. We predicted that the processes of integrating and updating reliable interdependent stable and dynamic sources of contextual information in the mid-phase of an action would be cognitively demanding, and so, we hypothesised a decrease in anticipation accuracy under dual-task conditions in the *Dependent* condition at this phase (Gredin et al., 2020b). In the final phase, the integration and updating of kinematic information with the contextual information was predicted to be underpinned by automatic processes (i.e. not elicit increases in cognitive load), thus we hypothesised that the secondary task would not interrupt these processes or impact performance (Runswick et al., 2018). Therefore, performance in the *Independent* condition would not be impacted by the secondary task in the final phase. However, in the *Dependent* condition, as increased task load was expected to impair the updating of stable contextual information with dynamic contextual information, it was predicted that the secondary task would detrimentally affect performance in the final phase as well (Gredin et al., 2020b).

## Method

### Participants

We conducted an a priori power analysis using the smallest effect size of interest approach in G*Power 3.1 (Faul et al., 2007; Lakens, 2022). For our repeated measures ANOVA to detect a small ($\eta_{p}^{2}$ = 0.02) within-subjects main effect for anticipation accuracy across the 18 measures (3 × 3 × 2) with a moderate correlation amongst repeated measures (r = 0.5) and a power of 0.8, a total of 28 participants would be required. Due to resource constraints and the availability of an appropriately skilled sample (Schweizer & Furley, 2016) we were able to recruit 25 skilled male soccer players (*M*_age_ = 21.28, *SD* = 3.55). However, to circumvent the potential issue of inflated effect sizes, we report adjusted effect size estimates (*d*_unbiased_, Cumming, 2012). Participants had a mean of 14.36 (*SD* = 4.42) years’ playing experience and all competed or had competed at a minimum of university level. At the time of the study, participants were taking part in a mean of 5.36 (*SD* = 2.66) hours’ soccer practice per week and at least one competitive soccer match per week. All participants reported having normal or corrected vision. Each participant provided written informed consent prior to taking part. This study was approved by the St Mary’s University Research Ethics Committee and the Brunel University Research Ethics Committee.

## Test stimuli

Thirty-six video sequences that represented two-versus-two defensive soccer scenarios were used as test stimuli. Sequences were filmed on a full-size AstroTurf soccer pitch using a high-definition digital video camera (Cannon XF100, Tokyo, Japan) with a wide-angle camera lens (Canon WD-H72 0.8x, Tokyo, Japan). To create footage that represented the first-person viewing perspective experienced by soccer players in competition, the camera was attached to a moving trolley at a height of 1.7 m. The camera (representing the defender that the participant took the role of) faced the other players moving towards the camera while moving backward to simulate the movement of the defender. Originally, a total of 130 video sequences were filmed and then edited using Adobe Premiere Pro video editing software (Adobe, CA, USA). However, based on two expert soccer coaches’ (qualified at UEFA A Licence level) ratings of the representativeness of the sequences, this number was reduced to 36 for use as experimental test stimuli.

Each sequence began with three players moving towards the camera: two attacking opponents and one defender who represented the participant’s teammate (see Fig. 1). The participant viewed the sequences from a first-person perspective as though they were a second defender. The opponent in possession of the ball began approximately 7 m from the camera and 3 m inside the halfway line and dribbled towards the defending team’s goal as the sequence progressed. The opponent off-the-ball and the on-screen defender began behind the opponent in possession on the right or left side, also moving towards the defending team’s goal. Approximately 1.5 s into the sequence, the opponent off-the-ball made an accelerated run that involved either staying on the side they began on or crossing over to finish on the other side of the opponent in possession, while the defender tracked this run by moving alongside him. Each sequence lasted 5 s, at which point the opponent in possession could move the ball to the left or right by either passing the ball to his teammate or dribbling in the direction opposite to where his teammate finished his run.

Each action sequence was temporally occluded at three distinct phases of the action to give three occlusion conditions named the *early* phase, *mid*-phase and *final* phase (see Fig. 1). For the early phase occlusion condition, the action was occluded after one second such that the final position of the opponent off-the-ball and reliable kinematic cues from the opponent in possession were not available. For the mid-phase occlusion condition, the action was occluded after three seconds such that the final position of the opponent off-the-ball was available. The opponent off-the-ball performed an accelerated run in the mid-phase and either stayed on the same side as they began the action or changed to the opposite side. Relevant kinematic cues to anticipate the opponent in possession’s final action were still not available. For the final phase occlusion condition, the action was occluded after five seconds just as the opponent in possession prepared to dribble or pass the ball, thus reliable kinematic cues were presented in this phase. Trials that concluded with a pass were occluded 120 ms prior to the last foot–ball contact from the opponent in possession, whereas trials that concluded with a dribble were occluded 240 ms before the opponent’s final foot–ball contact (cf. Gredin et al., 2020a). Occlusion conditions were predicated on prior work, observing that the time between the emergence of relevant kinematic information and the last moment of foot–ball contact was shorter when the last action was a pass versus a dribble (Gredin et al., 2018).

Within each temporal occlusion condition, each action sequence was also presented in three context conditions named the *Control* condition, *Dependent* condition and *Independent* condition. In the control condition, no stable contextual information was provided. In the experimental conditions, stable contextual information was explicitly provided as probabilistic information pertaining to either opponent action type preferences (i.e. Dribble = 67%, Pass = 33%) in the Dependent condition or opponent directional preferences (i.e. Right = 67%, Left = 33%) in the Independent condition. Participants were informed that, should the opponent dribble, they would always do so away from where the opponent off-the-ball is positioned at the end of the action sequence. Alternatively, should they pass, they would always do so in the direction of the opponent off-the-ball. This meant that the reliability of the action type preferences was dependent on the availability and monitoring of dynamic contextual information picked up from the movement of the opponent off-the-ball which occurred in the mid-phase of the action (e.g. Gredin et al., 2018, 2020b, 2020c). The reliability of the directional preferences, on the other hand, was independent of any form of dynamic contextual information and was thus reliable throughout the entire action (e.g. Broadbent et al., 2018). The proportion of actions was consistent across all three context conditions, but participants were only explicitly provided with directional and action type preferences in the Independent and Dependent conditions, respectively.

Finally, to increase the cognitive load under dual-task load conditions, participants were required to complete a secondary letter recall task. On these blocks, prior to each trial commencing, a series of four letters were presented on screen for 1 s each. Participants were required to memorise the series of letters and then recall them in the presented order after they provided their anticipation response on the primary task. To create the secondary letter recall task, 21 letters (all letters of the alphabet except vowels) were quasi-randomly generated to create four-letter series. A member of the research team ensured that all of the four-letter series were unique, that no recognisable words or common acronyms were used, and that no letter appeared more than once within a four-letter series. A total of 108 series of letters were therefore generated for use across the nine dual-task conditions.

Table 1 provides an overview of the occlusion, context and dual-task conditions employed in the experiment. The combination of conditions allowed for the reliability of information available to participants to be systematically manipulated. In the early phase, the only reliable information available was presented in the *Independent* condition where participants were provided with stable contextual information about the opponent’s directional preferences (i.e. % Right vs. Left). In the mid-phase, the opponent off-the-ball made their accelerated run to either side of the opponent in possession and, as such, the stable contextual information about the opponent’s action type preference (i.e. % Dribble vs. Pass) became reliable if integrated and updated with the dynamic contextual information from the position of the opponent off-the-ball. In the final phase, reliable kinematic information from the opponent in possession became available to use in all context conditions, but in the Independent and Dependent context conditions, participants could also integrate and update contextual information sources with the reliable kinematic information from the opponent in possession’s action. The dual-task conditions were hypothesised to supress the use of reliable contextual information compared to the single-task conditions.

## Apparatus and procedure

Test stimuli were presented on a standard-size laptop or personal computer. Participants were instructed to sit approximately 60 cm from the screen. 15 participants completed the experiment at a computer in the same room as one of the researchers. The remaining 10 participants completed the experiment on their own computer in the same ‘virtual’ room as one of the researchers via an online video conferencing platform (Zoom Video Communications, CA, USA) due to the COVID-19 pandemic which restricted face-to-face data collection. Only one participant was tested at a time.

Participants were tasked to verbally anticipate the final direction (left or right) of the ball upon occlusion of the footage. Prior to commencing the experiment, participants were first given an overview of the protocol. Participants then performed 10 familiarisation trials to become accustomed to the task requirements under the upcoming experimental conditions. Thereafter, participants completed 18 blocks of 12 condition-specific test trials. The 18 blocks comprised of a balanced combination of the occlusion (3 levels), context (3 levels) and task load (2 levels) conditions. Participants performed each occlusion condition (early phase, mid-phase and final phase) combined with each context condition (Control, Dependent, Independent) which created nine test blocks. Each occlusion × context condition was presented under two different task load conditions (single-task load, dual-task load) resulting in 18 test blocks in total.

**Fig. 2:**
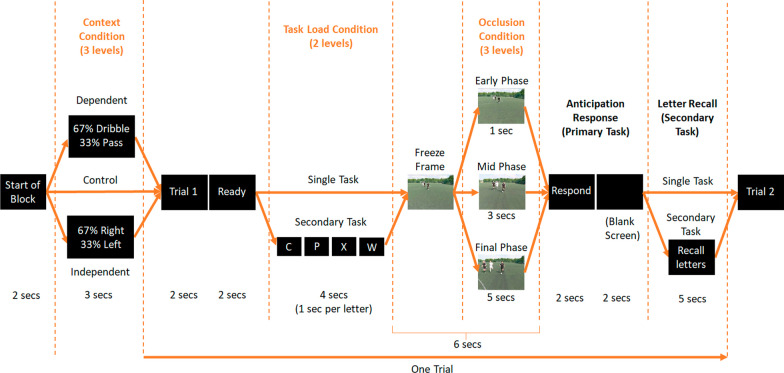
A schematic of the timeline (from left to right) for individual trials showing how the different occlusion, context and task load conditions were employed. Duration (in seconds) of each stage of a trial is presented below the schematic

A schematic overview of the protocol and the various combinations of conditions can be found in Fig. 2. Prior to each block commencing in the three context conditions, participants were either provided with no information (Control), information about opponent action type preferences (Dependent), or directional preferences (Independent), both verbally and on screen. The twelve trials within the block would then begin. Participants were first presented with a screen to ready themselves for the trial to begin. Next, depending on the task load condition, they were either presented with a series of four letters (in dual-task conditions) or the trial would move straight to the presentation of the action sequence (in single-task conditions). Each video of the action began with a freeze frame to allow participants time to determine the starting positions of the players and the ball, information that would normally be readily available in similar scenarios in a real-world setting (cf. Roca et al., 2011). This freeze frame was five, three and one second long for trials presented in the early, mid- and final phases, respectively. The video of the action would then commence, lasting one, three and five seconds in trials occluded at the early, mid- and final phases, respectively. This ensured that each video of the action, regardless of occlusion condition, lasted a total of six seconds. When the action occluded, participants responded by anticipating the direction the ball would be moved (left/right). In dual-task conditions only, participants then had five seconds to recall the series of four letters in the sequence they were presented at the beginning. A between trial interval of 4 s was employed (in which the trial number was presented, and participants were signalled to get ready) prior to the next trial commencing automatically.

The 36 trials originally selected for use in the experimental protocol were distributed across the 18 blocks. Each block was made up of 12 different trials with the proportion of right/left (Right = 67%, Left = 33%) and dribble/pass (Dribble = 67%, Pass = 33%) actions being consistent within each block. The order in which blocks were presented was counterbalanced across participants. To minimise the effect that the stable context conditions could have on performance in subsequent blocks, two ‘washout’ blocks were included after block 4 and block 9. In these blocks, participants were provided with directional (Right = 17%, Left = 83%) or action type (Dribble = 17%, Pass = 83%) preferences of the opponent that differed from those provided in the experimental trials, which were explicitly presented to participants prior to the blocks commencing. To further mitigate any potentially confounding learning and familiarity effects across conditions, no performance feedback was provided to participants at any point.

Upon completion of each block of trials, to ascertain the perceived usefulness of the various information sources, participants rated how useful they found a) the probabilistic information related to opponent preferences (stable contextual information), b) the information related to the opponent off-the-ball (dynamic contextual information) and c) the information related to the opponent in possession of the ball (kinematic information), on a Likert scale of 0 to 10, with 0 being not at all useful and 10 being extremely useful. Additionally, to ascertain the degree of cognitive load required to complete the task, participants completed three dimensions of the National Aeronautics and Space Administration-Task Load Index (NASA-TLX) Questionnaire (Hart & Staveland, 1988). Participants were required to rate from low to high (on a scale of 0 to 100) how mentally demanding and temporally demanding the task was, as well as from good to poor how successfully they felt they performed the task. The NASA-TLX was developed as a measure of mental workload that is not specific to tasks and conditions and was validated across roles in NASA (e.g. flight simulations and cognitive laboratory tasks). It has been used successfully to assess the demands of sporting tasks, both specifically when assessing the impact of contextual factors (Mullen et al., 2021) and in soccer-specific research (e.g. Auer et al., 2021; Filipas et al., 2021).

## Data processing

Z-scores and box plots were used to identify outliers for each variable. Values were considered to be outliers if they were more than three interquartile ranges above or below the upper and lower quartiles of the dataset, respectively. Six extreme outliers were identified in the anticipation accuracy data, three extreme outliers were identified when rating the usefulness of information, and finally, a single extreme outlier in rating of mental demand was identified. In each of these instances, the values were replaced with the mean of the remaining data in the condition in which the outliers were identified. In the case of violations of Mauchly’s test of sphericity, the Greenhouse–Geisser correction was applied. Alpha was set at 0.05. Partial eta squared ($\eta_{p}^{2}$) and Cohen’s *d*_unbiased_ were used to report effect size. In all instances in which multiple pairwise comparisons were conducted (e.g. to identify the cause of main effects), Bonferroni corrections were applied to account for familywise error. All analyses were conducted using IBM SPSS Statistics (version 26).

## Results

### Planned data analysis

#### Manipulation checks

We first conducted multiple manipulation checks. In the interest of brevity, the full outputs of the manipulation checks are in Additional file: 1. As a manipulation check for the use of contextual information in the experimental conditions compared to the control condition, we compared the perceived usefulness of the different information types (stable contextual information [probabilistic information related to opponent preferences], dynamic contextual information [movement/positioning of the opponent off-the-ball], kinematic information [opponent in possession]) across conditions. A 3 Information Type (Stable contextual information, Dynamic contextual information, Kinematic information) × 3 Occlusion (Early, Mid, Final) × 3 Context (Control, Independent, Dependent) × 2 Task Load (Single, Dual) repeated measures ANOVA was conducted on perceived usefulness of information sources. The manipulation of contextual information was successful, as the Independent (*M* = 5.92, *SD* = 2.54, *d*_unb_ = 1.70) and Dependent (*M* = 6.06, *SD* = 2.50, *d*_unb_ = 1.96) context conditions were perceived to contain more useful information than the Control condition (*M* = 3.98, *SD* = 3.59, both *p* < 0.01).

As a manipulation check for the additional task load in the dual-task conditions compared to the single-task conditions, a paired t-test was used to compare the effect of task load on the three dimensions of the NASA-TLX (i.e. mental demand, temporal demand, perceived performance). The manipulation of task load was successful, as mental demand was greater in the dual-task (*M* = 70.82, *SD* = 10.92) compared to the single-task conditions (*M* = 48.29, *SD* = 14.78, *p* < 0.01, *d*_unb_ = 1.68).

#### Secondary task performance

Performance on the secondary task was calculated as the percentage of letters recalled correctly, in the order they were presented, within each block. Participants recalled an average of 86.62% (*SD* = 9.65) of the letters correctly. To examine performance on the secondary task, a 3 Information Type × 3 Occlusion ANOVA was conducted on the percentage of letters recalled from dual-task conditions. The results of the 3 Occlusion × 3 Context ANOVA are presented in Table 2. ANOVA revealed no significant main effects of Occlusion or Context condition on secondary task performance, nor was the Occlusion × Context interaction significant.

**Table 2:** Secondary task (letter recall) performance (3 Occlusion × 3 Context ANOVA), primary task anticipation accuracy of ball direction (3 Occlusion × 3 Context × 2 Task Load ANOVA) and NASA-TLX mental demand (3 Occlusion × 3 Context ANOVA) statistical test results

Effect	*Df*	*F*	*P*	$\eta_{p}^{2}$
*Secondary Task Performance*				
Occlusion	2,48	.38	.69	.02
Context	2,48	.48	.62	.02
Occlusion × Context	4,96	1.54	.20	.06
*Primary Task Anticipation Accuracy*				
Occlusion	2,48	193.46	< .01	0.89
Context	2,48	3.39	.04	0.12
Task Load	1,24	4.53	.04	0.16
Occlusion × Context	4,96	1.39	.24	0.06
Occlusion × Task Load	1.55,37.28	1.93	.17	0.07
Context × Task Load	2,48	.76	.47	0.03
Occlusion × Context × Task Load	4, 96	2.15	.08	0.08
*NASA-TLX Mental Demand*				
Occlusion	1.17,48	24.69	< .01	0.51
Context	2,48	4.69	.01	0.16
Occlusion × Context	3.05,73.10	0.94	.43	0.04

#### Primary task anticipation accuracy

For the primary task, anticipation accuracy was calculated as the percentage of trials on which the correct direction (left/right) was anticipated. Anticipation accuracy was calculated in this way irrespective of performance on the secondary task. Before conducting the analysis, the data collected in person was compared to the data collected online, and for the primary task, overall anticipation accuracy was not significantly different between the two conditions, (*p* = 0.71). Therefore, the two datasets were collapsed to allow for the analysis of the full participant sample.

**Fig. 3:**
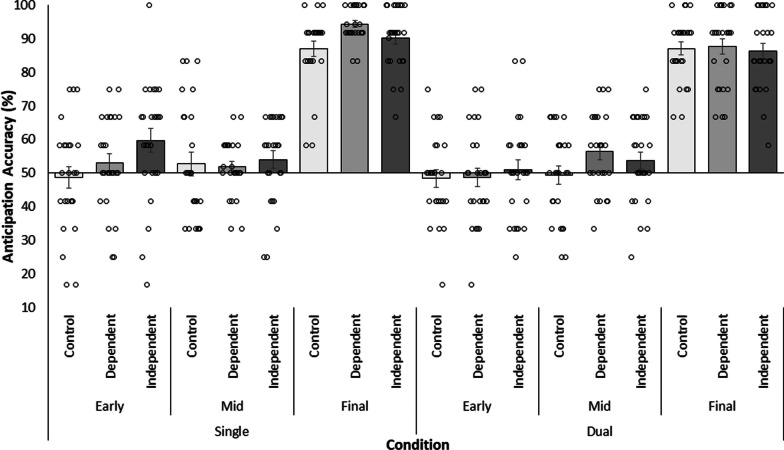
Mean anticipation accuracy of ball direction in the primary task across task load condition (Single, Dual), occlusion condition (Early, Mid, Final phase) and context condition (Dependent, Independent) (bars) with standard error and individual participant data points (dots). X axis crosses at chance

To determine how anticipation accuracy of ball direction in the primary task was affected across conditions, a 3 Occlusion (Early, Mid, Final) × 3 Context (Control, Independent, Dependent) × 2 Task Load (Single, Dual) repeated measures ANOVA was conducted. Mean anticipation accuracy scores (and SE) (percentage of trials on which participants correctly judged the final direction of the ball [left/right]) are presented in Fig. 3. The results of the repeated measures ANOVA are presented in Table 2. First, ANOVA revealed a significant main effect of Occlusion condition on anticipation accuracy of ball direction. Anticipation accuracy was higher in the final phase (*M* = 88.77%, *SD* = 10.15) of the action sequence compared with the early (*M* = 51.56%, *SD* = 15.4, *p* < 0.01, *d*_unb_ = 4.71) and mid-phase (*M* = 52.97%, *SD* = 13.03, *p* < 0.01, *d*_unb_ = 4.87), with no differences being observed between these latter phases (*p* > 0.99, *d*_unb_ = 0.17). A significant main effect of Context on anticipation of ball direction was also observed. Participants exhibited higher anticipation accuracy when provided with Dependent (*M* = 65.30%, *SD* = 21.57, *p* = 0.13, *d*_unb_ = 0.47) or Independent contextual information (*M* = 65.82%, *SD* = 20.91, *p* = 0.08, *d*_unb_ = 0.55). However, while medium effect sizes were observed, the pairwise comparisons were not statistically significant. ANOVA also revealed a significant main effect of Task Load on anticipation accuracy. Participants were more accurate in judging ball direction in the single-task (*M* = 65.71%, *SD* = 22.07) than the dual-task (*M* = 63.15%, *SD* = 21.08) condition.

#### Mental demand

The results of the 3 Occlusion (Early, Mid, Final) × 3 Context (Control, Independent, Dependent) ANOVA for mental demand are presented in Table 2. The outputs for the other two variables from the NASA-TLX (temporal demand and perceived performance) are reported in Additional file 1: 1. The ANOVA revealed a significant main effect of Occlusion condition on mental demand. Participants reported higher levels of mental demand when viewing the early phase (*M* = 67.66, *SD* = 20.55) of the action sequence compared with the mid-phase (*M* = 61.13, *SD* = 19.76, *p* < 0.01, *d*_unb_ = 0.54) and final phase (*M* = 49.87, *SD* = 24.85, *p* < 0.01, *d*_unb_ = 1.18). A significantly higher level of mental demand was also reported in the mid-phase compared to the final phase (*p* < 0.01, *d*_unb_ = 0.77). A significant main effect of Context was observed. Participants reported higher mental demand in the Dependent (*M* = 62.19, *SD* = 21.85) compared with the Control (*M* = 56.90, *SD* = 24.44,* p* = 0.04, *d*_unb_ = 0.40) condition but no significant differences in mental demand were observed between the Dependent and the Independent (*M* = 59.57, *SD* = 22.47, *p* = 0.25, *d*_unb_ = 0.22) condition, or between the Independent and Control condition (*p* = 0.40, *d*_unb_ = 0.20).

## Exploratory data analysis

### Anticipation accuracy: congruence

The results of pre-planned analysis showed mixed findings on the positive effects of context at earlier occlusion periods, and this suggested that there may be a congruence effect in the data and that further exploratory analysis that separates congruent and incongruent trials could provide greater insight into our initial analysis. The congruence effect refers to the common finding that accuracy increases for actions congruent with the stable contextual information and decreases for incongruent ones (Gredin et al., 2020a). For example, should a soccer player receive stable contextual information that an opponent is more likely to move the ball to the right than the left (opponent directional preferences), and this information aligns with the opponent’s action (movement of the ball to the right), anticipation accuracy would be enhanced relative to if this contextual information had not been provided. However, when the stable contextual information does not align with the opponent’s action (movement of the ball to the left), anticipation accuracy is detrimentally affected (e.g. Loffing et al., 2015; Runswick et al., 2019). Gredin et al. (2020b) found that additional task load did not affect anticipation performance on congruent trials, but the performance of skilled soccer players decreased on incongruent trials under task load. The authors suggested that additional task load may impact the integration and updating of contextual information with conflicting kinematic information in the final phase of the trial.

Therefore, to determine the effect of congruence on anticipation accuracy of ball direction across the various conditions, we conducted a further 2 Congruence (Congruent, Incongruent) × 3 Occlusion (Early, Mid, Final) × 2 Context (Independent, Dependent) × 2 Task Load (Single, Dual) repeated measures ANOVA. Congruent trials were those in which the opponent in possession’s actions aligned with the contextual information whereas incongruent trials were those in which the opponent in possession’s actions conflicted with the opponent preferences. The Control condition was omitted from this analysis due to no probabilistic information being provided.

**Fig. 4:**
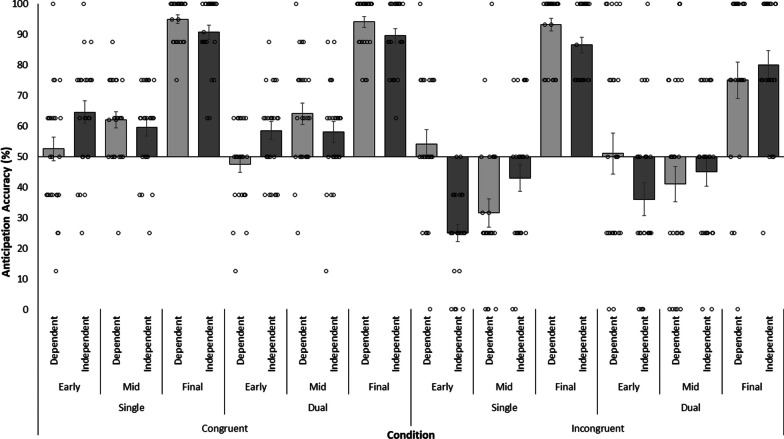
Mean anticipation accuracy of ball direction in the primary task across congruence (Congruent, Incongruent), task load (Single, Dual), occlusion (Early, Mid, Final phase) and context (Dependent, Independent) conditions (bars) with SE and individual data points (dots). X axis crosses at chance. Trials in the control condition were not included in this analysis

Mean anticipation accuracy scores (and SE) are presented in Fig. 4. The results of the 2 Congruence × 3 Occlusion × 2 Context × 2 Task Load repeated measures ANOVA are presented in Table 3. There was a significant main effect of Congruence, which showed participants anticipated ball direction more accurately on congruent (*M* = 69.62%, *SD* = 21.82) than incongruent (*M* = 55.10%, *SD* = 31.93) trials. A main effect of Occlusion condition on anticipation accuracy of ball direction was also observed. Anticipation accuracy was higher in the final phase (*M* = 87.96%, *SD* = 17.22) compared with the early (*M* = 48.63%, *SD* = 24.23, *p* < 0.01, *d*_unb_ = 4.50) or mid-phase (*M* = 50.50%, *SD* = 23.09, *p* < 0.01, *d*_unb_ = 4.71).

**Table 3:** Anticipation accuracy of ball direction: congruence (2 Congruence × 3 Occlusion × 2 Context × 2 Task Load ANOVA) statistical test results

Effect	*df*	*F*	*P*	$\eta_{p}^{2}$
*Anticipation Accuracy: Congruence*				
Congruence	1,24	36.40	< .01	0.60
Occlusion	2,48	179.66	< .01	0.88
Context	1,24	1.53	.23	0.06
Task Load	1,24	1.00	.33	0.04
Congruence × Occlusion	2,48	4.04	.02	0.14
Congruence × Context	1,24	3.23	.09	0.12
Occlusion × Context	2,48	2.04	.14	0.08
Congruence × Task Load	1,24	0.14	.71	0.01
Occlusion × Task Load	2,48	3.27	.047	0.12
Context × Task Load	1,24	0.47	.50	0.02
Congruence × Occlusion × Context	2,48	21.27	< .01	0.47
Congruence × Occlusion × Task Load	2,48	3.41	.04	0.12
Congruence × Context × Task Load	1,24	2.21	.15	0.08
Occlusion × Context × Task Load	2,48	3.09	.05	0.11
Congruence × Occlusion × Context × Task Load	2,48	1.71	.19	0.07

A significant Congruence × Occlusion interaction was observed. Anticipation accuracy was higher on congruent compared with incongruent trials, but the difference was larger in the mid-phase (congruent: *M* = 60.86%, *SD* = 15.45, incongruent: *M* = 40.13%, *SD* = 24.81, *p* < 0.01, *d*_unb_ = 1.58) than the early phase (congruent: *M* = 55.75%, *SD* = 17.45, incongruent: *M* = 41.50%, *SD* = 27.80, *p* < 0.01, *d*_unb_ = 1.22), which was in turn larger than the difference in the final phase (congruent: *M* = 92.25%, *SD* = 10.26, incongruent: *M* = 83.66%, *SD* = 21.29, *p* < 0.01, *d*_unb_ = 0.74). ANOVA also revealed a significant Occlusion × Task Load interaction. Anticipation accuracy of ball direction was higher under single- than dual-task conditions in the final phase (Single: *M* = 91.29%, *SD* = 11.11, Dual: *M* = 84.63%, *SD* = 21.24, *p* < 0.01, *d*_unb_ = 0.64) while no significant differences were observed in the mid-phase (Single: *M* = 48.99%, *SD* = 22.28, Dual: *M* = 52.00%, *SD* = 23.88, *p* = 0.21, *d*_unb_ = 0.32) or early phase (Single: *M* = 49.00%, *SD* = 23.88, Dual: *M* = 48.25%, *SD* = 24.68, *p* = 0.82, *d*_unb_ = 0.06).

A significant Congruence × Occlusion × Context interaction was also observed. Follow-up 2 Congruence × 3 Occlusion ANOVAs in each context condition revealed a two-way interaction in the Dependent, *F* (2,48) = 9.31, *p* < 0.01, $\eta_{p}^{2}$ = 0.28 and Independent, *F* (2,48) = 16.91, *p* < 0.01, $\eta_{p}^{2}$ = 0.41, conditions with different causes. In the Dependent condition, a significant congruence effect was observed (performance was higher on congruent and lower on incongruent trials) on trials in the mid-phase (congruent: *M* = 62.98%, *SD* = 15.35, incongruent: *M* = 36.26%, *SD* = 26.24, *p* < 0.01, *d*_unb_ = 1.51) and final phase (congruent: *M* = 94.44%, *SD* = 7.91, incongruent: *M* = 84.09%, *SD* = 23.95, *p* = 0.01, *d*_unb_ = 0.76), but not when only the early phase was viewed (congruent: *M* = 50.00%, *SD* = 16.56, incongruent: *M* = 52.50%, *SD* = 28.68, *p* = 0.62, *d*_unb_ = 0.14). Moreover, the difference was most pronounced in the mid-phase. Conversely, a congruence effect was observed for each of the Independent condition time points but the effect became progressively smaller from the early phase (congruent: *M* = 61.50%, *SD* = 16.53, incongruent: *M* = 30.50%, *SD* = 22.18, *p* < 0.01, *d*_unb_ = 2.03) to the mid-phase (congruent: *M* = 58.75%, *SD* = 15.41, incongruent: *M* = 44.00%, *SD* = 22.90, *p* < 0.01, *d*_unb_ = 0.97) and to the final phase (congruent: *M* = 90.06%, *SD* = 11.84, incongruent: *M* = 83.23%, *SD* = 18.49, *p* = 0.048, *d*_unb_ = 0.53).

Finally, a significant Congruence × Occlusion × Task Load interaction was observed. Follow-up 3 Occlusion × 2 Task Load ANOVAs were run to investigate the effect of these factors on anticipation accuracy of ball direction in congruent and incongruent trials. For congruent trials, only a main effect of Occlusion was observed, *F* (2,48) = 142.45, *p* < 0.01, $\eta_{p}^{2}$ = 0.86, as anticipation accuracy was significantly greater in the final phase (Single: *M* = 92.76%, *SD* = 9.99, Dual: *M* = 91.75%, *SD* = 10.60) compared to the early (Single: *M* = 58.50%, *SD* = 19.47, Dual: *M* = 53.00%, *SD* = 14.85, *p* < 0.01, *d*_unb_ = 4.42) and mid-phase (Single: *M* = 60.73%, *SD* = 13.60, Dual: *M* = 61.00%, *SD* = 17.45, *p* < 0.01, *d*_unb_ = 3.34). The difference in accuracy between the early and mid-phase was not significant (*p* = 0.14, *d*_unb_ = 0.50). Conversely, for the incongruent trials, a significant Occlusion × Task Load interaction was observed, *F* (2,48) = 4.15, *p* < 0.05, $\eta_{p}^{2}$ = 0.15. While anticipation accuracy was similar in single- and dual-task conditions in the early phase (Single: M = 39.50%, SD = 24.27, Dual: M = 43.50%, SD = 31.06, *p* = 0.47, *d*_unb_ = 0.20) and mid-phase (Single: M = 37.26%, SD = 23.17, Dual: M = 43.00%, SD = 26.26, *p* = 0.25, *d*_unb_ = 0.30), anticipation accuracy was negatively affected by the secondary task in the final phase (Single: M = 89.82%, SD = 8.71, Dual: M = 77.50%, SD = 26.37, *p* < 0.01, *d*_unb_ = 0.68).

## Discussion

The aim of the current study was to investigate how changes in information reliability and task load impacted the use of stable contextual information that is either *independent* of, or *dependent* on, evolving dynamic contextual information during a two-versus-two soccer anticipation task. The interdependency between the stable and dynamic contextual sources of information, and the amount of reliable kinematic information, was manipulated in a systematic manner using the temporal occlusion paradigm, and task load was manipulated using the dual-task paradigm (letter recall) to provide a unique insight into the processes underpinning anticipatory judgements in sport.

In line with previous research, compared to the Control condition, providing stable contextual information, which was either dependent on or independent of dynamic contextual information, tended to improve anticipation accuracy in the final phase (Broadbent et al., 2018; Gredin et al., 2018, 2020c). Furthermore, as predicted, anticipation accuracy improved as more reliable kinematic information became available in the final phase, compared to in the preceding phases (Müller & Abernethy, 2012; Runswick et al., 2020). These findings support the notion that athletes continually update and integrate information sources based on their relative reliability to predict the outcome with the highest likelihood of success (Knill & Pouget, 2004). Therefore, the current paper adds to the growing body of empirical evidence proposing Bayesian integration theory as a framework for anticipation in sport (e.g. Gredin et al., 2018, 2020c; Helm et al., 2020; Loffing & Hagemann, 2014).

While we made specific predictions about how the interdependency between information sources would yield differential effects across phases of the action sequence and dual-task conditions, our primary analysis did not reveal any significant interactions, despite mental demand being perceived to be highest when required to process *dependent* stable contextual information. However, exploratory analysis, which also considered congruence as an influencing factor, allowed us to explore the issue further. As expected, and in line with previous research (Gredin et al., 2018, 2020b), participants anticipated more accurately on congruent than incongruent trials, supporting the manipulation checks by showing participants were engaging with the stable contextual information provided. More specifically, a congruence effect was observed in the Independent condition in each occlusion condition, but in the Dependent condition the congruence effect was only observed in the mid-phase and final phase. This shows that the contextual information provided in the Independent condition was reliable and utilised in every phase. In contrast, but in line with our hypotheses, the contextual information provided in the Dependent condition was utilised in the mid-phase as well as in the final phase. This data indicates that participants in the mid-phase were actively using the dynamic contextual information related to the positioning of the opponent off-the-ball to update and utilise the stable contextual information in the form of opponent action preferences (Gredin et al., 2018, 2020b). Congruence effects were observed in all conditions in which reliable stable contextual information was available, but the size of the effects decreased over the course of the action sequence, suggesting that participants are likely to have assigned increased weight to more reliable kinematic cues as they emerged towards the end of the action sequence (Gredin et al., 2020c; Helm et al., 2020). Again, these findings highlight a continuous updating and integration of information sources based on their relative reliability (Harris et al., 2022; Knill & Pouget, 2004).

Anticipation accuracy on congruent and incongruent trials was differentially affected by the secondary (letter recall) task. On congruent trials, anticipation accuracy increased across occlusion conditions (Gredin et al., 2020c), and dual-tasking did not affect performance (Güldenpenning et al., 2020). On incongruent trials, where the stable contextual information conflicted with the outcome of the individual trial, the inclusion of a secondary task did not affect anticipation accuracy in the early or mid-phase of the action sequence but degraded performance in the final phase. Performance was therefore negatively affected by the secondary task when both contextual information and reliable opponent kinematics were available. This is likely due to the disruption caused by the secondary task on the integration and updating of contextual information with conflicting kinematic information (Gredin et al., 2020b).

Our findings suggest that updating contextual information by integrating kinematic information does place some demand on working memory in all instances, regardless of whether information is dependent on, or independent of, other contextual information sources. We had expected a detrimental effect of increased task load to be observed in conditions that required processing *dependent*, compared to *independent*, stable contextual information due to the higher number of probabilistic rules that the athletes had to process (Kaplan & Berman, 2010). However, in contrast to this and the findings by Runswick et al. (2018), our findings suggest that the process of updating and integrating contextual and kinematic information is not purely underpinned by automatic processes and does place some cognitive demand on working memory, irrespective of any interdependency between contextual information sources. It is important to note that the sport and the nature of contextual information available in the current study were different compared to the study by Runswick et al. (2018). For example, in the current study, explicit stable contextual information was provided in the form of probabilistic percentages. In contrast, Runswick et al. (2018) provided domain-specific contextual information such as game score and field positions, from which participants could assign probabilities to potential event outcomes. The ability to perceive information from such sources is likely to be developed through extensive experience and practice and may therefore be governed by more automatic processes, in comparison with novel probabilistic information that needs to be held in working memory during task performance (Ericsson & Kintsch, 1995).

The findings described in this study have a number of implications for practice where explicit contextual information is provided to performers. For example, in situations that do not allow the athlete to wait for reliable kinematic information to unfold before initiating a defensive action (e.g. under severe time constraints), the provision and use of stable contextual information can enhance performance but only when congruent with the action outcome (Gredin et al., 2018; Runswick et al., 2018). Furthermore, in situations where the athlete can wait for kinematic information to become reliable before acting (e.g. under lenient time constraints), additional task load (e.g. when required to simultaneously communicate with teammates) appears to have a detrimental effect on performance in the presence of stable contextual information related to opponent preferences. Specifically, anticipation performance seems to be negatively affected by additional task load in the presence of kinematic information that conflicts rather than aligns with stable contextual information (i.e. when there is incongruence between the contextual information and the to-be-anticipated action outcome). Awareness of this detrimental effect may facilitate coaches in forecasting the utility of priming players with stable contextual information in highly dynamic and cognitively demanding performance contexts, such as soccer. Future research should examine the best way to deliver stable contextual information, such as through exposure rather than explicit instruction (see Thomas et al., 2022), as well as test various approaches to train an athlete’s ability to update contextual information with kinematic information under cognitively demanding performance conditions.

These implications should be considered alongside some limitations of the current study. Data collection for this study was conducted using stimuli presented on a screen that was temporally occluded, requiring verbalised responses. While this is a commonly used approach across experimental psychology (e.g. Causer et al., 2017; Farrow et al., 2005), when recruiting and investigating individuals with specific expertise in a movement skill, such as soccer defending (e.g. Casanova et al., 2013; Vater et al., 2015), the findings can be diluted without the inclusion of a movement response or more realistic stimuli (Dicks et al., 2010). Building on the current study, future research may wish to design experiments involving more interactive stimuli to examine the replicability of the findings presented. Moreover, while we have been able to subjectively suggest that our findings provide support for the proposals of Bayesian integration theory as a framework for anticipation in sport, a more objective assessment of the extent to which this is true would be beneficial. Through the use of formal computational models, Harris et al. (2023) recently demonstrated that, in line with Bayesian models, when anticipating ball bounce direction in rugby, expert rugby players place decreasing weight on prior information and more weight on visual cues as actions progress and those cues become more reliable. It would be interesting to extend such research to ascertain the extent to which the effect of factors such as secondary tasks align, more objectively, with Bayesian principles.

Finally, it is worth noting that the test stimuli of 216 trials were created from 36 videos repeated across the test blocks, so we cannot rule out the potential for confounding learning or familiarity effects during the test session. However, to ‘balance out’ any learning or carry-over effects across conditions, the order in which conditions were presented was counterbalanced across participants. Furthermore, to reduce the risk of familiarity across reused trials, the test stimuli were not characterised by any trial-specific features besides those of relevance for the current study (i.e. whether the players off-the-ball were on the left or the right side of the opponent in possession and whether the opponent passed or dribbled to the left or the right). To further mitigate potential familiarity effects across conditions, no performance feedback was provided upon trial completion and the order in which trials were presented in each condition was randomised (cf. Thomas et al., 2022).

In conclusion, this study used a systematic approach to provide novel evidence for the impact of information reliability and task load on the integration and updating processes associated with the use of contextual and kinematic information during anticipation in sport. Skilled soccer players were shown to effectively integrate contextual and kinematic information to enhance anticipation, providing further support for proposing Bayesian integration theory as a framework for anticipation in sport (e.g. Gredin et al., 2020a; Harris et al., 2022). Detrimental effects of increased task load were confined to the final phase of the action sequence when participants were required to integrate contextual and kinematic sources of information, with this drop in performance occurring irrespective of any interdependency between the stable and dynamic contextual information sources. These findings have implications for the way information is provided, processed and utilised in domains that necessitate the integration of contextual factors with live sensory information under severe time constraints, such as sport.

### Supplementary Information

**Additional file 1**: Manipulation checks: statistical analysis of ratings of perceived usefulness of information sources, and NASA-TLX ratings. 

## Data Availability

Datasets and materials used are available from the corresponding author upon request.
