# Supplementary material for: The effect of task load, information reliability and interdependency on anticipation performance
Source: Cogn Res Princ Implic. 2024 Apr 14;9:22. doi: 10.1186/s41235-024-00548-8 (PMC11016527; doi:10.1186/s41235-024-00548-8)
Supplement: Supplementary file 1 — Additional file 1: Manipulation checks: statistical analysis of ratings of perceived usefulness of information sources, and NASA-TLX ratings. [file 41235_2024_548_MOESM1_ESM.docx]

**Ratings of Perceived Usefulness of Information Sources**

In the interest of brevity, only main effects of Information Type, Temporal Occlusion, and Context are reported as these verify that participants used the information provided at the desired timepoints. ANOVA revealed a significant main effect of Information Type on perceived usefulness, *F*(1.37,32.93) = 25.29, *p* < .01, $\eta_{p}^{2}$ = 0.51. Overall, participants rated the opponent on-the-ball (*M* = 6.11, *SD* = 2.85, *d*_unb_ = 1.53) and the opponent off-the-ball (*M* = 5.92, *SD* = 2.34, *d*_unb_ = 1.56) as being more useful sources of information than the stable contextual information (*M* = 3.90, *SD* = 3.44, both *p* < .01). A significant main effect of Temporal Occlusion was also observed, *F*(1.61,38.56) = 42.16, *p* < .01, $\eta_{p}^{2}$ = 0.64. There was deemed to be more useful information in the final phase (*M* = 6.03, *SD* = 3.16) than the mid phase (*M* = 5.32, *SD* = 2.84, *d*_unb_ = 0.75), in which there was deemed to be more useful information than in the early phase (*M* = 4.62, *SD* = 3.04, all *p* < .01, *d*_unb_ = 0.61). There was also a significant main effect of Context, *F*(1.34,32.07) = 69.63, *p* < .01, $\eta_{p}^{2}$ = 0.74, with the Dependent (*M* = 6.06, *SD* = 2.50, *d*_unb_ = 1.96) and Independent (*M* = 5.92, *SD* = 2.54, *d*_unb_ = 1.70) conditions deemed to contain more useful information than the Control condition (*M* = 3.98, *SD* = 3.59, all *p* < .01). No other significant main effects or interactions were observed.

**NASA-TLX Ratings**

**Dual-Task Effects.** As a manipulation check, we compared mental demand, temporal demand, and perceived performance under single and dual task conditions using repeated measures t-tests. Participants rated higher mental demand in dual-task (*M* = 70.82, *SD* = 10.92) than single-task conditions (*M* = 48.29, *SD* = 14.78, *t*[24] = 9.04, *p* < .01, *d*_unb_ = 1.68). Temporal demand was also perceived to be higher in dual task (*M* = 54.67, *SD* = 16.30) than single task (*M* = 38.07, *SD* = 17.57, *t*[24] = 6.08, *p* < .01, *d*_unb_ = 0.95) conditions. Finally, participants perceived lower levels of performance in the dual-task (*M* = 46.16, *SD* = 10.89) compared to the single-task conditions (*M* = 42.40, *SD* = 11.30, *t*[24] = 2.21, *p* = .04, *d*_unb_ = 0.33).

**Temporal Occlusion and Context Effects.** We further analysed the effect of Temporal Occlusion and Context Condition on temporal demand and perceived performance. A 3 Temporal Occlusion × 3 Context Condition Repeated Measures ANOVA revealed a significant main effect of Temporal Occlusion on perceived temporal demand, *F*(1.27,30.48) = 25.77, *p* < .01, $\eta_{p}^{2}$ = 0.52. Participants reported higher levels of perceived temporal demand in the early phase (*M* = 58.00, *SD* = 28.68) compared with the mid phase (*M* = 45.33, *SD* = 21.70, *d*_unb_ = 0.33), which in turn was deemed more temporally demanding than the final phase (*M* = 35.77, *SD* = 21.80, *d*_unb_ = 0.56, all *p* < .01). A significant main effect of Temporal Occlusion on perceived performance was observed, *F*(1.25, 29.96) = 38.70, *p* < .01, $\eta_{p}^{2}$ = 0.62. Participants perceived lower levels of performance in the early phase (*M* = 57.47, *SD* = 19.72) compared with the mid phase (*M* = 46.87, *SD* = 16.14, *d*_unb_ = 0.73), which was in turn lower than the final phase (*M* = 28.50, *SD* = 17.36, *d*_unb_ = 1.39, all *p* < .01). No other main effects or interactions were observed.
